# Contemporary damage control surgery outcomes: 80 patients with severe abdominal injuries in the right upper quadrant analyzed

**DOI:** 10.1007/s00068-017-0768-8

**Published:** 2017-02-27

**Authors:** M. Hommes, S. Chowdhury, D. Visconti, P. H. Navsaria, J. E. J. Krige, D. Cadosch, A. J. Nicol

**Affiliations:** 1 Department of Surgery, Trauma Centre, Groote Schuur Hospital, University of Cape Town, Cape Town, South Africa; 2 Department of Surgery, University of Cape Town, Cape Town, South Africa; 3 Department of Trauma Surgery, Leiden University Medical Centre, Leiden, The Netherlands; 4 Surgical Gastroenterology, Hepatopancreaticobiliary Unit, Groote Schuur Hospital, Cape Town, South Africa; 5 Triemli City Hospital Zürich, Birmendorfstrasse 497, 8063 Zurich, Switzerland

## Abstract

**Background:**

Damage control laparotomy (DCL) is a well-established surgical strategy in the management of the severely injured abdominal trauma patients. The selection of patients by intra-abdominal organs involvement for DCL remains controversial. The aim of this study was to assess the injury to the abdominal organs that causing severe metabolic failure, needing DCL.

**Methods:**

Severely injured abdominal trauma patients with a complex pattern of injuries were reviewed over a 52-month period. They were divided into DCL and definitive repair (DR) group according to the operative strategy. Factors identifying patients who underwent a DCL were analyzed and evaluated.

**Results:**

Twenty-five patients underwent a DCL, and 55 patients had DR. Two patients died before or during surgery. The number and severity of overall injuries were equally distributed in the two groups of patients. Patients who underwent a DCL presented more frequently hemodynamically unstable (*p* = 0.02), required more units of blood (*p* < 0.0001) and intubation to secure the airway (*p* < 0.0001). The onset of metabolic failure was more profound in these group of patients than DR group. The mean Basedeficit was − 7.0 and − 3.8, respectively, (*p* = 0.003). Abdominal vascular (*p* = 0.001) and major liver injuries (*p* = 0.006) were more frequently diagnosed in the DCL group. The mortality, complications (*p* < 0.0001), hospital (*p* < 0.0001), and ICU stay (*p* < 0.009) were also higher in patients with DCL.

**Conclusion:**

In severely injured with an intricate pattern of injuries, 31% of the patients required a DCL with 92% survival rate. Severe metabolic failure following significant liver and abdominal vascular injuries dictates the need for a DCL and improves outcome in the current era.

## Introduction

Damage control laparotomy (DCL) is useful for a subset of abdominal trauma patients. The patients with gunshot wounds to the abdomen and significant blunt abdominal trauma who present with hemodynamic instability, acidosis, and coagulopathy are likely to benefit from a DCL [1–5]. This approach resulted in improved survival of critically injured and shocked patients based on the retrospective case series and when compared with historical controls (Table 1). However, there is concern about the lack of research relating to the indications and timing for a DCL [6].

**Table 1:** Criteria for Damage Control Laparotomy in patients who sustained blunt abdominal trauma or abdominal gunshot wounds

Criteria for DCL
Complex pattern of injuries [4, 5, 7, 8]
Operating time for DR of injuries > 60–90 min [7–9]
Initial hypothermia: *T* < 35 °C [10–13]
Initial acid base status: pH < 7.2; BE < 10–15; lactate < 5 mmol/L [12–16]
Non-surgical bleeding, onset of coagulopathy [17–20]
Transfusion requirements > 10 units packed red cells [18, 19, 21–23]

*DCL* Damage control laparotomy, *DR* definitive repair, *T* temperature, *BE* base excess

The liver is the most commonly injured organ following abdominal trauma [24]. The mortality associated with severe isolated hepatic injury is 10% which reaches up to 70% with associated three or more major organ injury [25, 26]. An early decision is crucial to initiate a DCL after rapid assessment of internal injuries and before severe metabolic failure has set in [27]. But concern has been expressed about identifying patients who might benefit from a damage control approach and patients who could tolerate definitive repair (DR) of the injuries [28, 29]. An appropriate selection for DCL is critical to decreasing morbidity, and unnecessary use of hospital facilities and expenses.

We compared two groups of patients with major abdominal injuries who were selected for a DCL and who were treated with DR of injuries. The aim of this study was to assess the injury to the abdominal organs causing severe metabolic failure, needing DCL.

## Methods

Major abdominal trauma was defined as two or more organs injured in the right upper quadrant (RUQ) of the abdomen in patients with an injury severity score (ISS) of >15 [30] and abbreviated injury score (AIS) (Abdomen) of ≥3 [31]. These patients were identified from a prospective trauma database during September 2008 to December 2012 at a level 1 trauma centre of Groote Schuur Hospital and included in the study for retrospective analysis. Patients with a single injury to the RUQ, ISS of <15, AIS < 3 or patients who died during surgery were excluded.

### Outcome

The primary outcome was survival to discharge. The secondary outcome was morbidity defined as general, and organ-specific complications, duration of intensive care (ICU), and hospital stay in days. Complications were graded by using the Clavien-Dindo grading system for the classification of surgical complications [21].

### Grading of injuries

Intra-abdominal injuries were graded according to the Organ Injury Scale of the American association of surgery for trauma (AAST) [32]. High-grade of injuries were considered to be grade 3 to 5.

### Operative management

Following an initial resuscitation according to the principles of the Advanced Trauma Life Support (ATLS®) [33], the physiological parameters were documented. Potential candidates for a DCL were non-responders to shock management, hypothermia, onset of metabolic failure, or a combination of these. Metabolic failure was defined as worsening metabolic acidosis (Base deficit), with or without coagulopathy (non-mechanical bleeding). Indications for surgery were hemodynamic instability, peritonitis or CT findings suggestive of bowel injury requiring surgical repair.

Operative management included DR of injuries or DCL. It was based on the institutional and definitive surgical trauma care (DSTC®) guidelines [34]. A DCL was defined as a limited operation for control of hemorrhage and contamination, secondary resuscitation in the ICU and DR during a reoperation. The decision to perform or to convert to a DCL was based on the preoperative physiological status, the severity of abdominal injuries and estimated time for repair of intra-abdominal injuries exceeding total operating time >60–90 min. Massive fluid resuscitation, a decrease in Base deficit after hemorrhage control, and the use of inotropes to improve hemodynamics were indications for conversion to a damage control strategy.

When severe shock, hypothermia, acidosis, and massive transfusion have led to coagulopathy and diffuse non-mechanical bleeding, the intra-abdominal cavity was packed. Patients with intra-abdominal packing were managed with an open abdomen.

Emergency reoperation was undertaken for the development of abdominal compartment syndrome or failure to attain the endpoints of resuscitation due to continuous hemorrhage. Treatment of complications was multidisciplinary when appropriate and included endovascular, endoscopic, and interventional CT or ultrasound guided drainage.

### Statistics

Results were presented as number (%) or as IQR. Patient groups were compared using the Pearson’s chi-squared test or Fisher’s exact test for categorical variables, and the Mann–Whitney test for non-normally distributed data. Statistical analysis was performed using statistical software (SPSS Inc, Chicago, IL,version 20). *P* values of <0.05 were considered to be statistically significant.

## Results

**Fig. 1:**
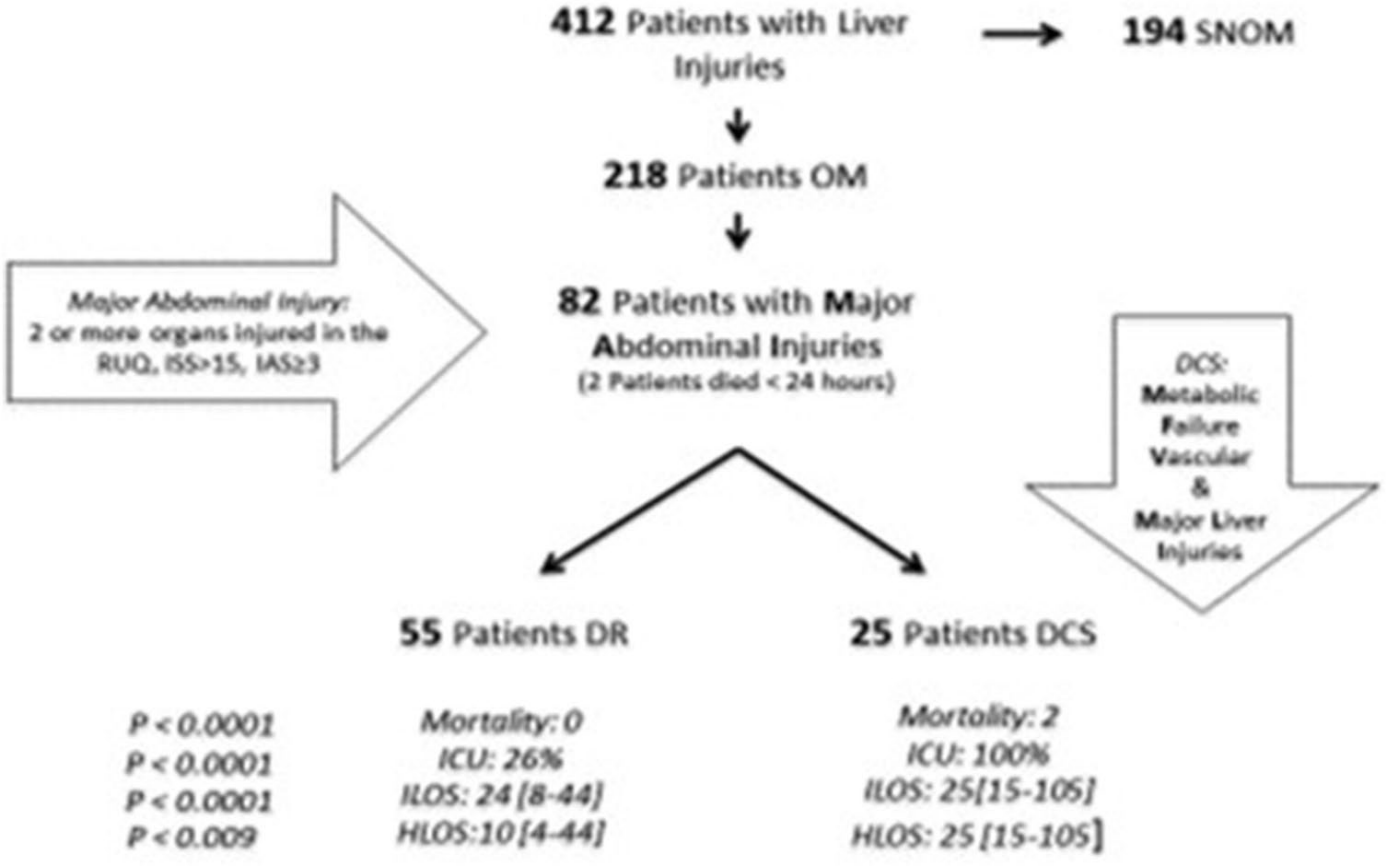
Management flowchart patients with abdominal trauma and a concomitant liver injury. *SNOM* Selective non-operative management, *OM* operative management, *RUQ* right upper quadrant, *ISS* injury severity score, *AIS* abdominal injury score, *DCS* damage control surgery, *DR* definitive repair, *ILOS* intensive care unit length of stay, *HLOS* hospital length of stay

Four hundred and twelve patients were diagnosed with a liver injury following RUQ abdominal trauma during the study period. One hundred and ninety-four patients were selected for non-operative management. Two hundred and eighteen patients with a liver injury underwent surgery. Eighty-two (38%) patients with a complex pattern of injuries were identified. Figure 1 presents a management flowchart of all patients with abdominal trauma and a concomitant liver injury.

Two patients died during or before the operation and were excluded for further analysis.

Eighty patients (Men 73, women 7, the mean age of 26 with a range of 13–57 years) who survived more than 24-h were included and further analyzed. Eleven (14%) patients sustained blunt trauma and 69 (86%) penetrating, of which 7 (10%) and 62 (90%) sustained stab wounds and gunshot wounds, respectively. The median ISS was 21 (IQR 16–32).

In 80 patients, 108 high-grade of injuries in the RUQ of the abdomen were diagnosed, liver (46), extrahepatic biliary tract (2), major vascular (12), right kidney (26), duodenum (10), and pancreas (12). Other associated intra-abdominal injuries diagnosed were stomach (21), diaphragm (15), small bowel (26), colon (17), spleen (13), left kidney (13), ureter (5), bladder (4), vascular (10), and pelvic fractures (4).

Thirty-four (42.5%) patients had isolated abdominal injuries. Forty-six (58%) patients sustained injuries in body regions other than the abdomen, included head and neck (9), face (5), thorax (36), and extremities (18).

The indications for surgery were hemodynamic instability in 17 (21%) patients, an acute abdomen in 56 (70%) patients, and 7 (9%) patients had CT findings of intra-abdominal injuries that required surgical repair.

Fifty-five (69%) patients had DR of their injuries, and 25 (31%) patients underwent a DCL.

The operative procedures in 25 patients who underwent a DCL are presented in (Table 2), and the postoperative general and organ-specific complications are presented in (Table 3).

**Table 2:** Number of surgical procedures in 25 patients who underwent a damage control laparotomy

Surgical procedures	*N*
Perihepatic packing	20
IVC packing	4
Drainage laceration of the common bile duct	2
Kidney packing	3
Duodenal primary repair	3
Nephrectomy	6
Infrarenal IVC ligation	2
Distal pancreatectomy	3
Colon ligation	5
Small bowel ligation	1

*IVC* Inferior vena cava

**Table 3:** aHundred and four surgical complications occurred in 25, complications classified according Clavien-Dindo classification

Grading of complications	Number of complications
I	18
II	29
IIIa	11
IIIb	10
IVa	25
IVb	7
V	4

I: Any deviation from the normal postoperative course

II: Requiring pharmacological treatment with drugs

IIIa: Requiring surgical, endoscopic, or radiological intervention not under general anesthesia

IIIb: Requiring surgical, endoscopic, or radiological intervention under general anesthesia

IVa: Life-threatening complication requiring ICU-management with single-organ dysfunction

IVb: Life-threatening complication requiring ICU-management with multiple-organ dysfunction

V: Death of a patient

### The magnitude of injuries

The higher ISS, major abdominal vascular injuries, and more high-grade liver injuries were diagnosed in patients who underwent a DCL (Table 4).

**Table 4:** General patient`s characteristics and magnitude of injuries

	DR	DCL	*P* value
	*N* = 55 (69%)	*N* = 25 (31%)	
Sex, *N* (%)			
M	51 (93%)	22 (88%)	0.67
F	4 (7%)	3 (12%)	
Age in years	25	30	0.03
Mechanism, *N* (%)			
Blunt	7 (13%)	4 (16%)	0.73
Penetrating	48 (87)	21 (84%)	
Gunshot wound	42/48 (87%)	20/21 (95%)	0.43
Stab wound	6/48 (13%)	1/21 (5%)	
Injury severity score	19	26	0.002
High-grade liver injury, *N* (%)	26/55 (47%)	20/25 (80%)	0.006^a^
Abdominal vascular injury, *N* (%)	9 (16%)	13 (52%)	0.001^b^
Extrahepatic biliary tree injury, *N* (%)	3 (5%)	2 (8%)	1.00
Pancreatic injury, *N* (%)	20 (36%)	11 (44%)	1.00
Duodenal injury, *N* (%)	14	5	1.00
Right kidney injury, *N* (%)	28	10	0.45
Bowel injury, *N* (%)	22 (40%)	11 (44%)	0.74
Abdominal injuries, *N* (%)			
3 organs	14 (25%)	2 (8%)	0.16
4 organs	11 (20%)	9 (36%)	
5 organs	17 (31%)	6 (24%)	
>5 organs	13 (24%)	8 (32%)	

*DR* Definitive repair, *DCL* damage control laparotomy, *N* = Number

^a^Odds ratio 4.46 (95% confidence interval 1.47–13.59)

^b^Odds ratio 5.54 (95% confidence interval 1.92–16.00)

### The physiological status

Patients who required a DCL presented more often with hypotension, required more frequently intubation to secure the airway and had received more units of blood products transfusion. The DCL group also had more profound metabolic acidosis than DR group (Table 5).

**Table 5:** Physiological parameters in 80 patients with severe abdominal trauma comparing patients undergoing DR versus DCL

	DR	DCL	*P* value	Odds ratio
	*N* = 55 (69%)	*N* = 25 (31%)		(95% CI)
Blood pressure < 90 mmHg on admission, *n* (%)	3 (5)	6 (24)	0.02	5.47 (1.24–24.10)
Intubation on admission, *n* (%)	8 (15)	16 (64)	<0.0001	10.44 (3.45–31.65)
Glascow Coma Scale ≤ 8 on admission, n (%)	1(2)	3 (12)	0.09	7.36 (0.73–75.69)
Hemoglobin in gm/dl, mean (SD)	11 (2)	10 (3)	0.06	
pH, mean (SD)	7.34 (0.09)	7.28 (0.08)	0.01	
Lactate in mmol/L, mean (SD)	2.6 (2.1)	3.9 (2.8)	0.03	
Base deficit, mean (SD)	−3.8 (4.0)	−7.0 (4.9)	0.003	
Metabolic failure (base excess ≤−5), *n* (%)	20 (36)	17 (68)	0.009	3.72 (1.36–10.15)
Blood transfusion *n* (%)	18 (33%)	21 (84%)	<0.0001	10.79 (3.22–36.14)
Units of blood, median, range	0 (0–7)	4 (0–12)	<0.0001	

*DR* Definitive repair, *DCL* damage control laparotomy, *n* number, *SD* standard deviation, *CI* confidence interval

### Outcome

Patients who underwent a DCL had an increased mortality (8% vs. 0%), more postoperative general, liver-related and duodenal complications. Hospital stay and the number of patients requiring ICU and ICU stay were also higher in patients who had a DCL (Table 6).

**Table 6:** Morbidity in 80 patients undergoing DR versus DCL

Morbidity	DR [*N* = 55 (69%)]	DCL [*N* = 25 (31%)]	*P* value
Patients with general complications	27 (49%)	24 (96%)	<0.0001^b^
Hospital stay in days	10 (4–44)	25 (15–105)	<0.0001^b^
Patients requiring ICU	14 (26%)	25 (100%)	<0.0001^c^
ICU stay in days	24 (8–44)	25 (15–105)	0.009^b^
Mortality	0 (0%)	2 (8%)	0.10^a^

*DR* Definitive repair, *DCL* damage control laparotomy

^a^Data were analyzed with a Pearson Chi-squared analysis

^b^Fisher’s exact test

^c^Mann–Whitney test

### Deaths

Two patients died during hospital stay (at day 12 and day 15). The first patient was a 35-year-old male who sustained multiple gunshot wounds (abdominal, groin and buttocks and extremities). This patient had a Gr V liver, and right kidney injury. A nephrectomy was performed, and the bleeding from liver was controlled with packing. Despite the control of surgical bleeding, this patient developed severe abdominal sepsis and required multiple relook laparotomies. Eventually, this patient died due to multi-organ failure on day 15.

The second patient was a 23-year-old male who sustained an abdominal gunshot wound and precordial stab. This patient had an open skull fracture and thoracoabdominal injury. An exploratory laparotomy and sternotomy were performed. A cardiac injury, diaphragm injury, grade 5 liver injury, pancreatic and gastric injury were identified. Despite the control of bleeding with packing, this patient developed abdominal sepsis and died due to multi-organ failure on day 15 of postinjury.

## Discussion

Definitive organ repair cannot be undertaken safely in a patient with a critical physiological status. These patients are more likely to die from their intra-operative metabolic failure than they are from the failure to complete organ repairs. Hypotension on admission, intubation on admission, requiring more units of blood transfused during resuscitation and presenting with a severe metabolic acidosis, abdominal vascular and high-grade liver injuries dictated the need for a damage control strategy in patients with major abdominal trauma evaluated in our study. Since the introduction of damage control surgery, it has been accepted that patients with severe injury and physiological derangements are selected for a DCL [3–5, 26, 35]. On the other hand, DCL should not be performed in patients who can tolerate DR of the injuries, causing an increase in morbidity and subsequent increase in the use of hospital facilities and costs [27, 28].

In liver trauma, packing has been a well-accepted surgical technique to control bleeding [26]. In patients with a complex pattern of injuries, control of bleeding is essential, and the severity of trauma and physiological derangements influence the decision to pack and delay definitive organ repair. The first step is the recognition of patients in the resuscitation room likely to need a DCL. The second step is to perform an exploratory laparotomy and to make a quick decision whether the patient needs a DCL or can tolerate DR. After control of bleeding, a rapid assessment to classify the severity of trauma and estimate the time required for definitive repair. At this stage, timing to initiate DCL is depending on physiological status or metabolic derangement. Previous studies have demonstrated that changes in core temperature, acidosis, and coagulation are essential, and initial preoperative temperature, P^H^, BE, transfusion requirements, and hemodynamic status are also important to make a decision for DCL (Table 1).

The role of postoperative angiography described in this study is limited. Due to an active surgical management policy with ligation of visible vessels in case of liver trauma, rendered early postoperative angiography rarely necessary. In this study, postoperative angiography was not performed routinely. *Although many arterial bleeders are deep in parenchyma and do not manifest clearly at laparotomy other authors recommend as the appropriate strategy to proceed with a postoperative angiography in the angiosuite after DCL for complex liver injury* [26].

Although there is no consensus on a validated definition of “severely injured” patients, in this study, we defined patients who sustained a complex pattern of injuries involving three or more organs in the RUQ of the abdomen with AIS > 3, and ISS > 15 as severely injured [17].

This study was performed in a busy level 1 trauma centre. The rate of DCL in this group of patients was 31% that is much higher comparing to the 6–18% described in the literature [36]. We did not feel we over triaged patients requiring a damage control laparotomy. The reason for a higher rate is most likely due to the selection of patients who sustained major abdominal trauma to the RUQ. The overall mortality in patients undergoing DCL was 8%. In the literature, the mortality rates for DCL varies from 26 to 67% [17]. Mortality following penetrating abdominal trauma is 10%, whereas mortality following severe blunt abdominal exceeds 40% [24]. Due to high interpersonal violence in Cape Town, the majority (84%) of the patients present with penetrating abdominal trauma than blunt trauma. It may explain a lower overall mortality rate in our study comparing with the literature. However, all patients who were selected for DCL and reached to the operating room had an 92% survival.

While the number of patients in this prospective series of severely injured patients with a complex injury pattern is low, comparison of small groups in this paper using significance testing needs to be interpreted in the light of the very low power to detect statistically significant differences. A clinical interpretation and familiarity with surgical strategies and techniques taught in the DSTC® or similar course have to be considered during comparisons and not just a statistical description.

While an increase in the incidence of patients who undergo DCL has been noted, we should be aware of the rise in morbidity in patients who unnecessarily suffer a DCL. Despite reports of increased survival after the introduction of DCL and implementation of a damage control strategy in the field of emergency surgery [1, 2], few authors conclude that evidence that supports the safety and efficacy of damage control is limited [36]. They call for the need of randomized controlled trials (RCT). An RCT would be confronted with the same dilemma, at the first overuse of DCL in patients who could also tolerate DR, or vice versa an increase in mortality or morbidity in patients who are selected for DR.

In conclusion, the current study did focus on criteria for selection of patients with severe abdominal injuries in the right upper quadrant of the abdomen who might benefit from DCL. 31% of the severely injured patients with a complex pattern of injuries required a DCL with 92% survival rate. A moderate onset of metabolic failure or hypotension on arrival is not a precise indications to perform a DCL. The onset of severe metabolic failure following major liver and abdominal vascular injuries dictates the need for a DCL with improved outcomes in the current *era*.
